# Estimated Risk of Radiation-Induced Cancer after Thymoma Treatments with Proton- or X-ray Beams

**DOI:** 10.3390/cancers13205153

**Published:** 2021-10-14

**Authors:** Anders Lideståhl, Gracinda Johansson, Albert Siegbahn, Pehr A. Lind

**Affiliations:** 1 Department of Oncology-Pathology, Karolinska Institutet, 17177 Stockholm, Sweden; 2 Department of Oncology, Södersjukhuset, 11883 Stockholm, Sweden; gracinda.johansson@regionstockholm.se (G.J.); albert.siegbahn@regionstockholm.se (A.S.); 3 Department of Clinical Science and Education, Karolinska Institutet, Södersjukhuset, 17177 Stockholm, Sweden; pehr.lind@regionstockholm.se

**Keywords:** radiation-induced secondary malignant neoplasm, organ equivalent dose, proton beam therapy, thymoma, thymic carcinoma

## Abstract

**Simple Summary:**

Thymic tumors, i.e., thymomas and thymic carcinomas, are rare tumors that derive from the remnant of the thymus gland. Although surgery is the first treatment of choice, some patients will be treated with radiotherapy. For many patients the prognosis is good, hence it is important to avoid treatment related complications such as radiation-induced secondary malignancies. Radiotherapy can be delivered with different techniques and with different particles. In the present study, we compare the calculated (estimated) risks for secondary malignancies after treatment of thymic tumors with two different photon (x-ray) radiotherapy techniques or with proton beam therapy. We use a commonly used radiobiological model to calculate the risks for radiation induced secondary malignancies for each treatment modality. In conclusion, proton beam therapy was shown to provide the potential for reducing the risk of secondary malignancies, compared to photon radiotherapy, after treatment of thymic tumors.

**Abstract:**

We compared the calculated risks of radiation-induced secondary malignant neoplasms (SMNs) for patients treated for thymic tumors with 3D-CRT, IMRT, or single-field uniform dose (SFUD) proton beam therapy (PBT) using the pencil beam scanning (PBS) technique. A cancer-induction model based on the organ equivalent dose (OED) concept was used. For twelve patients, treated with 3D-CRT for thymic tumors, alternative IMRT and SFUD plans were retrospectively prepared. The resulting DVHs for organs at risk (OARs) were extracted and used to estimate the risk of SMNs. The OED was calculated using a mechanistic model for carcinoma induction. Two limit cases were considered; the linear-exponential model, in which the repopulation/repair of the cells is neglected, and the plateau model, in which full repopulation/repair of the irradiated cells is assumed. The calculated risks for SMNs for the different radiation modalities and dose-relation models were used to calculate relative risks, which were compared pairwise. The risks for developing SMNs were reduced for all OARs, and for both dose-relation models, if SFUD was used, compared to 3D-CRT and IMRT. In conclusion, PBS shows a potential benefit to reduce the risk of SMNs compared to 3D-CRT and IMRT in the treatment of thymic tumors.

## 1. Introduction

Thymomas, i.e., tumors that derive from the epithelium of the thymus gland, are the most common tumor of the anterior mediastinum [1]. The etiology is unknown, although the diagnosis seems to be associated with autoimmune diseases such as Myasthenia Gravis [2]. Thymic tumors may be non-invasive (WHO Type A thymoma) or show loco-regional invasive, or even potentially metastatic traits (WHO Type B thymomas and Type C thymic carcinomas, respectively) [3]. Surgery is the first treatment of choice; however, as positive surgical margins are common, and some tumors are unresectable, postoperative radiotherapy (RT) (PORT) or definitive RT are indicated for many patients, often in combination with concomitant chemotherapy [4,5]. The tumor location adjacent to vital organs at risk (OARs), e.g., lungs, heart, and esophagus, makes it a rather challenging diagnosis in RT. Most patients treated for thymoma have a good long-term prognosis [6], hence it is vital to avoid late radiation-induced toxicity and secondary malignant neoplasms (SMNs) as much as possible. Most patients are treated with photon beam RT delivered by three-dimensional conformal RT (3D-CRT) or intensity-modulated RT (IMRT) [7]. Due to better target dose conformity obtained with IMRT, the higher doses given to the OARs are usually given to smaller volumes compared to 3D-CRT. However, the integral dose given to the entire body is often significantly higher in IMRT, and this may cause more SMNs compared to 3D-CRT [8,9].

Previous clinical and planning studies have demonstrated the possibility to reduce the predicted rates of toxicity and SMNs if proton beam therapy (PBT) is used in the treatment of thymomas, instead of photon RT [10,11,12,13,14]. However, most studies focused on the use of PBT with the passive scattering technique. In the present study, we focused on the use of active-scanning PBT to reduce SMNs, compared to photon beam RT techniques, i.e., 3D-CRT and IMRT.

Schneider et al. introduced the concept of organ equivalent dose (OED) for implementation in models to assess the risk of SMNs when comparing different treatment plans or different treatment modalities [15]. The OED concept assumes that any inhomogeneous three-dimensional dose distribution in an organ corresponds to an OED which, when given homogeneously to the organ, results in the same rate of radiation-induced cancer. This concept considers two parameters, viz., the organ specific cancer incidence rate at low dose, and the organ specific cell-sterilization at high doses. In the present study, based on the concept of OED, the predicted rates of SMNs in patients treated for thymoma with 3D-CRT were compared to those predicted with alternative dose plans, prepared for IMRT and PBT. In our estimations we assume both a linear-exponential and a plateau curved dose-response relationship for the risk of induction of SMNs [8].

## 2. Materials and Methods

### 2.1. Patient, Tumor, and Treatment Characteristics

Twelve patients that previously received external photon beam therapy with 3D-CRT for thymic tumors at our department were included in this study.

Patient, tumor, and treatment characteristics have been described in a previous study [14] and are presented in Table 1. Median age was 64 years (range: 49–82 years) and ten patients were male. Thymectomy had been performed in 9 patients. For all patients, computed tomography (CT) images were acquired, which were subsequently used for treatment planning. The CT images included the patient anatomy from the patient’s chin and neck to the abdomen. All patients were treated with 3D-CRT as neoadjuvant, PORT, or definitive RT. For all patients, photon plans with IMRT and single-field uniform dose (SFUD) spot-scanning PBT were retrospectively prepared. The plan comparison in terms of the dose-volume values for the target volumes and the relevant organs at risk (OAR), together with the estimated normal tissue complication probability (NTCP), have previously been reported [14].

### 2.2. Treatment Planning

The treatment planning for photon- and proton-beam therapy have been described in a previous article [14]. Fractionation doses and target volumes are presented in Table 1. Median target dose was 56.0 Gy (range 50.0–60.0 Gy). PTV coverage for all plans were set at 95% to 107% of the prescribed dose. Treatment planning for 3D-CRT and IMRT plans were performed on the Eclipse™ treatment planning system (TPS) version 13.7 (Varian Medical Systems, Palo Alto, CA, USA). The 3D-CRT plans were prepared with 3 to 5 incident photon beams (6, 15, and 18 MV energies) delivered by a Varian linear accelerator. The IMRT plans were prepared with 5 coplanar photon beams (6 MV) and delivered by a Varian linear accelerator. The dose-volume constraints set for the OARs in the optimization of the IMRT plans, were as follows: spinal cord: Dmax ≤ 48 Gy (RBE), total lung (-CTV): V20 ≤ 35%, esophagus: Dmean ≤ 45 Gy (RBE). For the heart, attempts were made to keep the dose as low as possible.

The planning for PBT was completed using spot-scanning beams with kinetic energies between 60 and 230 MeV, generated in an IBA machine (Ion Beam Applications, S.A., Louvain-La-Neuve, Belgium). The plans were prepared using the single field uniform dose (SFUD) method. To ensure dose coverage of the part of the PTV which was located closer to the patient surface, a range shifter of water equivalent thickness of 3.5 g/cm^2^ was used. The treatment planning constraints used for 3D-CRT and IMRT planning were also adopted for the SFUD planning. A two-beam configuration was used for proton irradiation. The field-specific PTV (fsPTV) tool, available in the Eclipse™ TPS, was then used for spot positioning. To account for the relative biological effectiveness (RBE) of proton beams, a generic RBE of 1.1 was assumed [10]. Dosimetric comparison between the 3D-CRT, IMRT, and SFUD plans are presented in Table 2. The resulting dose-volume histograms (DVH) for the OARs were extracted and used to estimate the risk of radiation-induced secondary cancers for the three treatment modalities studied.

### 2.3. Calculation of Risk of Radiation-Induced Secondary Cancers

The risk for developing SMNs following RT of thymic cancers was calculated using a radiation-induction cancer-model developed by Schneider and co-authors [15,16]. This model is based on the concept of organ equivalent dose (OED) [15], for which it is assumed that any inhomogeneous three-dimensional dose distribution in an organ corresponds to the same OED which, when given homogeneously to the organ, results in the same rate of radiation-induced cancer.

The relationship between dose and the risk of developing cancer is linear up to doses of around 2 Gy [17]. Above this dose threshold value, the dose-risk dependence deviates from linearity due to the sterilization of already mutated cells, which then becomes important [15]. The organ-specific cancer risk incidence is given by Equation (1),

(1) $$I^{org}=I_{0}^{org}\times OED^{org}$$

where $I_{0}^{org}$ is the excess absolute cancer risk (cancer incidence rate) at low doses, derived from epidemiological studies. In the present study, the values of $I_{0}^{org}$ used for the calculation of secondary cancers were taken from UNSCEAR 2000 report [18] for the organs esophagus, breast (the two breasts were considered as one organ), skin, lungs (total lung excluding the part overlapping with the clinical target volume (CTV)), thyroid, and stomach (Table 3). We also included in our calculations the estimation of other solid cancers, the OAR for the “Other solid” was considered all the volume encompassed by the computed tomography (CT) image, excluding all other considered OARs and the CTV (Table 2). The value of the cancer incidence rate at low doses for other solid tumors was taken from the study by Schneider and collaborators [15]. The OED was calculated using a mechanistic model for carcinomas [16,19], which accounts for cell killing and that the radiation dose is delivered in fractionation regimen, Equation (2).

(2) $$OED^{org}=\frac{1}{V}\sum_{i}DVH\left(D_{i}\right)\cdot \frac{e^{-\alpha^{\prime }D}}{\alpha^{\prime }R}\left(1-2R+R^{2}e^{\alpha^{\prime }R}-{\left(1-R\right)}^{2}e^{-\frac{\alpha^{\prime }R}{1-R}D}\right)$$

In Equation (2), the parameter *R*, with a value in the interval 0–1, characterizes the repopulation/repair of the irradiated tissue between two treatment fractions. *R* takes a value of 0 if no repopulation/repair is considered and a value of 1 for full repopulation/repair between treatment fractions. Similar to the study by Schneider et al. [15], in the present study two limit cases were considered when applying the cancer model. One of these was the linear-exponential model, in which the repopulation/repair of the cells is neglected, Equation (3),

(3) $$OED^{org}=\frac{1}{V}\sum_{i}DVH\left(D_{i}\right)\cdot D_{i}\cdot e^{\alpha^{\prime }\cdot D_{i}}$$

The other limit case of the mechanistic model, the plateau model, considers that there is a full repopulation/repair of irradiated cells, Equation (4),

(4) $$OED^{org}=\frac{1}{V}\sum_{i}DVH\left(D_{i}\right)\cdot \frac{\left(1-e^{\alpha^{\prime }\cdot D_{i}}\right)}{\alpha \prime }$$

In Equations (2)–(4), $DVH\left(D_{i}\right)$ is the volume receiving dose $D_{i}$ in the *DVH*, $\alpha^{\prime }=\alpha +\beta d$ where α and β are the linear and quadratic parameters of the LQ-model describing the cell kill following irradiation.

### 2.4. Statistical Analysis

All data were tested for normality and were found to be non-normally distributed. Therefore, a 2-sided Wilcoxon signed-rank test was used for pair-wise comparisons. A *p*-value of < 0.05 was considered statistically significant. All data was tested pairwise for each patient, treatment modality, and dose-response model.

## 3. Results

Results are presented in Table 2, Table 4, Table 5, Table 6 and Table 7. As previously reported [14], target doses were comparable between treatment plans. Doses given to all OAR were significantly reduced in the PBS plans compared to the 3D-CRT and IMRT plans, with the exceptions of esophagus (Dmax), breast (Dmax), and skin (Dmax). Doses to the lungs and stomach were significantly increased in the IMRT plans compared to 3D-CRT, however, there were no significant difference between these modalities in the doses produced in the other OARs (Table 2).

The OED, the excess absolute risk (EAR), and the relative risk for developing SMNs were significantly reduced in the PBS plans for all OARs, and for both dose-relation models, compared to the 3D-CRT and IMRT plans (Table 4, Table 5 and Table 6). The overall risk of SMNs was significantly reduced in the SFUD plans compared to 3D-CRT and IMRT for all individual patients (Table 7). IMRT posed a higher risk for developing SMNs compared to 3D-CRT for most OARs and for both dose-relation models.

## 4. Discussion

In the present study, we have confirmed previous findings that PBT significantly reduces the predicted risk of SMNs compared to photon-based RT. In our study, we compared PBT to both 3D-CRT and IMRT and, as expected, IMRT resulted in higher rates of predicted SMNs compared to 3D-CRT for almost all OARs studied. In the calculation of the risk of SMNs two dose-relation models were used, viz., the linear-exponential and the plateau model. With both models, significant reductions in predicted risks for SMNs were calculated in favor of PBT. The plateau model produced higher risks of SMNs throughout all OARs, independently of treatment modality. Since the plateau model, as opposed to the linear-exponential model, considers full repopulation of the irradiated cells such a result was anticipated. As suggested by Schneider et al., the real risk for SMN probably lies somewhere between these scenarios [9].

In photon RT, the primary radiation field contributes the most to the rate of radiation-induced SMNs, although scattered x-rays and secondary neutrons also contribute to this risk [9]. Accordingly, all sources of radiation during RT must be considered to determine the risk of SMNs. However, the role of the primary dose for the risk SMNs tends to become reduced when higher photon energies are used, i.e., higher MV beams results in more scattered x-rays and more neutrons which are causing more SMNs [9].

Intensity-modulated RT allows for better dose contouring around the target and lower doses to OARs compared to 3D-CRT. However, these advantages come at the cost of low dose exposure to larger volumes, i.e., higher integral dose, than is the case for 3D-CRT. This “low-dose bath” is caused by the larger number of treatment fields and longer beam-on time, i.e., additional monitor units (MUs), that are typically required in most IMRT plans compared to 3D-CRT [22]. The beam-on time is increased as the MLC blocks part of the beam, which may also contribute to scattered x-rays [23]. In theory, IMRT should therefore typically cause lower rates of acute and late toxicity and higher rates of SMNs compared to 3D-CRT. The choice to use IMRT in the clinic is normally based on the high degree of dose conformity to the target volume of the high-dose isodose-lines achievable with this method. It can be expected that the presence of high doses outside of the target volume will be the most important factor to predict the risk of early and late side-effects other than SMNs. For younger patients with a good prognosis, the risk of SMNs becomes a more important issue, which may need to be considered when selecting treatment method. Secondary-cancer induction models that account for a linear dose-response relationship at low doses and a plateau-shaped dose-response relationship at high doses, due to cell-sterilization of already mutated cells, will always favor 3D-CRT before IMRT, due to the low-dose bath seen in IMRT [17]. However, recent epidemiological studies have cast a doubt whether the risk for SMN really is higher in IMRT compared to 3D-CRT [24,25]. In a study by Xiang et al., the risks for secondary cancers were compared in 450 thousand patients registered in the National Cancer Database who received treatment with 3D-CRT, IMRT, or PBT for different tumors between 2004 and 2015. The risk of a second cancer was similar in 3D-CRT and IMRT, whereas PBT was associated with a lower risk compared to IMRT [24]. Clinical findings may support a linear dose-response relationship in SMN induction even at high doses, which would implicate that the low-dose bath seen in IMRT is not as important as previously assumed [26]. Additionally, updated secondary-cancer models incorporating epidemiological data have been proposed [27,28].

The most common PBT techniques in use are passive scattering (often called double scatter (DS)-PBT), and active scanning (often called pencil beam scanning PBT (PBS)) [29,30]. In the passive scattering method, the treatment beam is formed by passing the beam through high-atomic number scattering foils, apertures, and range compensators, which shapes the proton beam to fit the shape of the target volume. When the protons hit the scatterers, secondary neutrons are formed which may contribute to the rate of SMNs. In PBS, the pencil beam is directed by a magnetic scanner without the use of beam modifying hardware, secondary neutrons are only produced inside the patient. Therefore, the dose produced by secondary produced neutrons and the resulting cancer risks are low in PBS-based PBT compared to passive-scattering PBT. An evaluation of neutron doses in PBS-based PBT and the associated risks for SMNs was performed in a study by Schneider et al. [31]. The neutron doses produced in PBS-based PBT was found to be at least 10 times lower than measured doses in passive-scattering PBT. Furthermore, when considering the total doses produced by the primary beam and by the generated neutrons, it was found that the total fatality risk from SMNs could be increased by a factor two with photon RT compared to PBS-based PBT. Scanning also allows for better dose shaping around the target volume than scattering, with the possibility of reducing doses to OARs [32]. The full advantage of protons regarding SMNs is thus reached primarily with the use of PBS [9].

Several studies concerning the risk for SMNs in patients treated with RT for thymoma have been published. A few groups have analyzed data from the Surveillance, Epidemiology, and End Results (SEER) program with somewhat contradictory results. Travis et al., presented data for 815 thymoma patients, registered between 1973 and 2000, treated with surgery alone or with RT [33]. The excess number of secondary cancers per 10,000 patients per year was significantly higher in the group treated with RT compared to the surgery alone group (74 vs. 49, *p* < 0.05). Some observations in this study are of interest; first, secondary cancers were also common among patients not treated with RT, second, many secondary cancers developed within a timeframe (≤ 4 years) that is not typical of radiation-induced SMN, and third, secondary cancers outside of the thorax was not uncommon. These observations, which indicate that thymoma may pose a risk for secondary cancers, is further enhanced in a SEER database study by Weklser et al., in which the incidence of extrathymic malignancies was found to be almost 20-fold in thymoma patients compared to general SEER population [34]. In that study, RT was not a significant risk factor for extrathymic malignancies. Fernandes et al., analyzed 1334 thymoma patients from the SEER database registered between 1973 and 2005 [35]. They found no significant differences in the risk for secondary malignancies after treatment with or without RT. Finally, Mou et al., analyzed 2234 thymoma patients from the SEER database registered between 1998 and 2013 who received surgery with or without PORT [36]. The proportion of patients presenting secondary malignancies was significantly higher in the group receiving PORT (12.2% vs. 9.4%, *p* = 0.034).

A couple of dose planning studies calculating the risk of SMN in thymoma patients have also been published. Franceschini et al., compared the risks produced with two novel techniques, volumetric modulated arc therapy (VMAT) and intensity-modulated proton therapy (IMPT), using photons and spot-scanning protons, respectively, in 20 patients with stage II–III thymoma [12]. The risk for secondary malignancies was calculated for breast, lungs, and esophagus using the so-called full model, as suggested by Schneider et al., which accounts for repopulation/tissue recovery between fractions [21]. The calculated EAR (per 10,000 patient-years) for induction of SMNs was significantly reduced for all three OARs in favor of IMPT compared to VMAT. Vogel et al., compared proton therapy plans with IMRT plans in 10 pts with stage II thymoma treated with adjuvant DS-PBT, using the OED model for calculation of risk of SMNs [11]. The predicted overall excess risk of SMNs (per 10,000 patients per year) was significantly reduced with PBT compared to IMRT (2.8 vs. 17.3, 95% CI 6.6–22.4). The rate of SMNs was lower than in the study by Travis et al., a fact that the authors attributed to inclusion of only stage II disease, lower treatment doses, and use of more modern techniques in their study compared to the older study. In our study, the predicted rates of SMNs were higher than in the study by Vogel et al. This may be attributed to higher prescribed doses in our study (median 56.0 vs. median 50.0 Gy). In their discussion, Vogel et al. suggested that PBS may be superior to DS-PBT in reducing doses to OAR, and thus bring down the risk for SMNs. They also suggested it would be particularly important for the thyroid, which received the same mean dose with DS-PBT and IMRT in their study. In our study, the mean dose to the thyroid was significantly reduced with PBS compared to both photon modalities, which corresponded to a significantly reduced predicted risk for SMNs in the thyroid with PBS. The conclusions of this work could be included in the clinical decision-making basis in the choice of radiotherapy method. Additional aspects that may influence the rate and importance of SMNs in RT patients, such as the age at irradiation and prognosis, should also be taken into consideration in such decisions.

There is only limited data published on the incidence of RT-induced secondary cancers after treatments using proton beams. In a short-term follow-up study involving 558 patients that received PBT, this cohort was compared with another cohort of the same size treated with photon RT [37]. A lower incidence of secondary cancer was observed for patients that received proton RT (5.2%), compared to patients that underwent photon RT (7.5%). The use of radiobiological models to predict risks for radiation-induced secondary malignancies could provide important information which can be used in the choice of RT modality for specific patient groups.

However, one should be cautious when using cancer risk models derived from treatments using photon-based therapy to predict risks associated with other treatment modalities such as PBT, especially as the carcinogenic effect of particles such as protons and neutrons are less known than for photons [38]. As more patients with thymoma and other intrathoracic tumors are treated with protons, and registered in SEER and similar databases, we may anticipate studies that differentiate the subsequent risk for SMNs between alternative treatment modalities.

A limitation of the present study is the low number of included patients. There is also a variation in the size of the target volume among these patients, which is responsible for the difference in the treatment-field configurations used. This inter-patient variability reflects a typical variation in the size of treatment volumes found in RT of thymic tumor patients. In the context of comparing between different RT modalities regarding SMNs, the intra-patient pairwise comparison provides means for evaluation of the different RT modalities for individual patients.

## 5. Conclusions

Our study confirms the potential benefit of PBT, delivered with scanned proton beams, regarding the possibility to reduce the risk of SMNs compared to 3D-CRT and IMRT in the treatment of thymic tumors.

## Figures and Tables

**Table 1:** Patient, tumor, and treatment characteristics.

Pt	Sex	Age (Years)	Surgery (y/n)	Margin	Chemotherapy	Histology (WHO)	RT Fractionation (Gy) (RBE)	CTV (cm^3^)	PTV (cm^3^)
1	M	76	n	-	carboplatin/etoposide	^1^	2 × 30	554	834
2	F	65	y	R1	none	B3	2 × 28	290	574
3	M	64	y	R0	none	C	2 × 28	318	756
4	M	63	y	R1	none	B3	2 × 28	170	400
5	F	59	y	R1	none	B2/3	2 × 28	257	525
6	M	56	n	-	cisplatin/docetaxel	C	2 × 30	305	596
7	M	53	y	R0	none	B3	2 × 27	132	451
8	M	52	y	R1	cisplatin/etoposide	B2/3	2 × 28	115	311
9	M	49	y	R1	cisplatin/etoposide	B3	2 × 28	153	369
10	M	73	n	-	carboplatin/etoposide	B3	1.8 × 30	80	498
11	M	82	y	R1	none	B2/3	2 × 30	130	351
12	M	74	y	R0	carboplatin/etoposide	C	2 × 25	760	1246

^1^ Biopsy showed infiltrative thymoma, no further classification.

**Table 2:** Dosimetric comparison between 3D-CRT, IMRT and PBT plans. Median values and the values of the first and third quartile are also presented.

	Parameter	3D-CRT	IMRT	SFUD	*p*-Value		
					3D-CRT vs. IMRT	3D-CRT vs. PBT	IMRT vs. PBT
CTV	D98 (%)	97.0 (96.3–98.0)	98.7 (97.1–99.4)	99.0 (98–99.8)	**	*	**
	D2 (%)	110.0 (110.0–111.0)	104.8 (104.6–105.0)	106.0 (105–107.8)	*	*	*
Spinal Cord	Dmax (Gy)	40.2 (17.3–42.4)	27.0 (25.2–33.3)	3.2 (0.3–12.0)	**	*	*
Oesophagus	Dmean (Gy)	20.2 (11.8–24.3)	17.9 (14.4–22.6)	7.6 (1.5–18.9)	**	*	*
	Dmax (Gy)	53.5 (41.3–57.0)	53.6 (47.7–58.4)	53.8 (31.3–59.6)	**	**	**
	V30 (%)	41.5 (13.5–47.5)	24.2 (16.1–38.2)	11.8 (0–32.2)	**	*	*
Heart	Dmean (Gy)	9.3 (1.7–11.6)	8.8 (2.0–10.6)	1.5 (0.2–6.7)	**	*	*
	V5 (%)	25.0 (2.3–40.3)	28.0 (3.6–36.1)	7.5 (1.0–17.2)	**	*	*
	V30 (%)	9.0 (0–16.8)	6.2 (0.0–13.5)	1.3 (0–10.6)	*	*	*
	V45 (%)	3.5 (0–9.5)	2.0 (0.0–9.1)	0.4 (0–6.6)	**	*	*
Lung (total)-CTV	Dmean (Gy)	13.4 (10.8–16.1)	14.7 (13.2–16.7)	5.8 (4.7–8.7)	*	*	*
	V5 (%)	55.5 (47.3–62.8)	60.1 (54.5–71.2)	20.0 (16.0–33.8)	*	*	*
	V20 (%)	22.0 (17.0–29.3)	31.3 (23.8–35.2)	10.5 (8.3–15.8)	*	*	*
Skin	Dmax (Gy)	46.9 (40.5–54.6)	44.4 (35.5–53.4)	50.6 (48.2–52.6)	**	**	**
Breast (total)	Dmean (Gy)	15.6 (10.2–30.4)	15.4 (13.7–20.5)	8.6 (5.5–12.8)	**	*	*
	Dmax (Gy)	51.7 (49.3–58.6)	54.7 (45.8–56.2)	51.2 (47.5–55.4)	**	**	**
Stomach	Dmean (Gy)	0.4 (0.2–0.6)	0.5 (0.3–0.6)	0 (0–0)	*	*	*
Thyroid	Dmean (Gy)	4.3 (1.8–17.7)	4.8 (1.9–17.6)	0.6 (0.1–9.9)	**	*	*

* *p* < 0.05, ** *p* > 0.05.

**Table 3:** Values of the parameters used for cancer risk estimations for all considered OARs.

OAR	$I_{0}^{org}\;(\mathrm{per}\;10,000\;\mathrm{Patients}/\mathrm{Year}/\mathrm{Gy}){\;}^{†}$	$\alpha \;{\left({\mathrm{Gy}}^{\;-1}\right)}^{\;‡}$	α/β (Gy)
Esophagus	0.63	0.0708	3
Breast	4.36	0.0670	3
Skin	0.58	0.0474	2.3
Lungs	8.27	0.0637	3.8
Thyroid	0.06	0.0251	3
Stomach	6.15	0.0959	10
Other solid	29.7	0.0800	3

^†^ Taken from UNSCEAR 2000 [18] for all OARs, except for other solid for which the value was taken from Schneider et al. [15]. ^‡^ Taken from Kehwar [20], except for breast for which the value was taken from Schneider et al. [21].

**Table 4:** OED (Gy). Median values and the values of the first and third quartile are also presented.

.		Solid Tumor		Skin		Esophagus		Lung		Breast		Stomach		Thyroid	
Source	Dose Response Relation	L-E ^1^	P ^2^	L-E	P	L-E	P	L-E	P	L-E	P	L-E	P	L-E	P
3D-CRT		0.87 (0.69–1.10)	1.88 (1.43–2.25)	1.15 (1.01–1.62)	1.69 (1.48–2.79)	1.47 (1.03–1.90)	5.62 (4.86–6.13)	2.21 (2.08–2.29)	5.23 (4.60–6.16)	1.71 (1.55–2.73)	6.36 (3.70–8.99)	0.37 (0.22–0.51)	0.38 (0.23–0.54)	2.71 (1.66–3.73)	3.34 (1.75–8.17)
IMRT		1.13 (0.87–1.33)	2.22 (1.76–2.78)	1.39 (1.24–1.91)	2.06 (1.78–3.08)	1.60 (1.19–1.80)	5.54 (5.21–6.60)	2.33 (2.17–2.53)	5.93 (5.51–6.93)	2.44 (2.03–2.85)	7.07 (5.58–8.85)	0.44 (0.27–0.56)	0.46 (0.27–0.59)	3.15 (1.71–3.85)	3.95 (1.80–8.39)
SFUD		0.14 (0.08–0.18)	0.39 (0.21–0.44)	0.18 (0.14–0.30)	0.54 (0.33–0.83)	0.60 (0.46–0.81)	2.44 (0.91–4.22)	0.72 (0.60–1.25)	2.06 (1.65–3.29)	0.84 (0.62–1.05)	2.90 (1.95–3.83)	0.01 (0.01–0.01)	0.01 (0.01–0.01)	0.49 (0.10–3.47)	0.53 (0.10–5.91)
Relative Reduction															
3D-CRT vs. IMRT		−0.26	−0.34	−0.24	−0.37 *	−0.13 *	0.08 *	−0.12	−0.70	−0.73	−0.71 *	−0.07	−0.08	−0.44	−0.61 *
3D-CRT vs. SFUD		0.73	1.49	0.97	1.15	0.87	3.18	1.49	3.17	0.87	3.46	0.36	0.37	2.22	2.81
IMRT vs. SFUD		0.99	1.83	1.21	1.52	1.00	3.10	1.61	3.87	1.60	4.17	0.43	0.45	2.66	3.42

^1^ Linear-Exponential model, ^2^ Plateau model, * *p* > 0.05, otherwise *p* < 0.05.

**Table 5:** Cancer risks (excess absolute risk given in /10,000 patients/year/Gy). Median values and the values of the first and third quartile are also presented.

		In-Field Total (-CTV) ^3^		Solid Tumor		Skin		Esophagus		Lung		Breast		Stomach		Thyroid	
Source	Dose Response Relation	L-E ^1^	P ^2^	L-E	P	L-E	P	L-E	P	L-E	P	L-E	P	L-E	P	L-E	P
3D-CRT		56.45	136.11	25.75 (20.36–32.70)	55.78 (42.54–66.97)	0.67 (0.58–0.94)	0.98 (0.86–1.62)	0.92 (0.65–1.20)	3.54 (3.06–3.86)	19.20 (18.13–19.94)	45.54 (40.06–53.63)	7.47 (6.78–11.90)	27.71 (16.14–39.18)	2.28 (1.37–3.15)	2.36 (1.39–3.34)	0.16 (0.10–0.22)	0.20 (0.11–0.49)
IMRT		69.21	156.08	33.58 (25.85–39.64)	65.91 (52.21–82.54)	0.81 (0.72–1.11)	1.20 (1.03–1.79)	1.01 (0.75–1.14)	3.49 (3.28–4.16)	20.31 (18.92–22.01)	51.63 (47.91–60.31)	10.62 (8.87–12.42)	30.81 (24.33–38.57)	2.69 (1.65–3.45)	2.80 (1.69–3.66)	0.19 (0.10–0.23)	0.24 (0.11–0.50)
SFUD		14.57	43.98	4.13 (2.28–5.31)	11.54 (6.18–13.02)	0.11 (0.08–0.17)	0.31 (0.19–0.48)	0.38 (0.29–0.51)	1.54 (0.58–2.66)	6.22 (5.23–10.92)	17.89 (14.35–28.65)	3.67 (2.72–4.59)	12.64 (8.49–16.73)	0.03 (0.03–0.04)	0.03 (0.03–0.04)	0.03 (0.01–0.21)	0.03 (0.01–0.35)
Excess SMNs																	
3D-CRT vs. IMRT		−12.76	−19.97	−7.83	−10.13	−0.14	−0.22 *	−0.09 *	0.05 *	−1.11	−6.09	−3.15	−3.10	−0.41	−0.44	−0.03	−0.04 *
3D-CRT vs. SFUD		41.88	92.13	21.62	44.24	0.56	0.67	0.54	2.00	12.98	27.65	3.80	15.07	2.25	2.33	0.13	0.17
IMRT vs. SFUD		54.64	112.10	29.45	54.37	0.70	0.89	0.63	1.95	14.09	33.74	6.95	18.17	2.66	2.77	0.16	0.21

^1^ Linear-Exponential model, ^2^ Plateau model, ^3^ presented as the sum of risks for all OARs, including solid tumor minus the CTV. * *p* > 0.05, otherwise *p* < 0.05.

**Table 6:** Relative risk of SMNs.

OAR	Relative Risk of SMNs					
	SFUD/3D-CRT		SFUD/IMRT		3D-CRT/IMRT	
	L-E ^1^	P ^2^	L-E	P	L-E	P
In-field total(-CTV)	0.26	0.32	0.21	0.28	0.82	0.87
Solid tumor	0.16	0.21	0.12	0.18	0.77	0.85
Skin	0.16	0.32	0.14	0.26	0.83	0.82 *
Esophagus	0.41	0.44	0.38	0.44	0.91 *	1.01 *
Lung	0.32	0.39	0.31	0.35	0.95	0.88
Breast	0.49	0.46	0.35	0.41	0.70	0.90 *
Stomach	0.01	0.01	0.01	0.01	0.85	0.84
Thyroid	0.19	0.15	0.16	0.13	0.84	0.83 *

^1^ Linear-Exponential model, ^2^ Plateau model. * *p* > 0.05, otherwise *p* < 0.05.

**Table 7:** Total risk of SMNs (excess absolute risk per 10,000 patients /year/Gy) for each patient, for each treatment modality, and dose response relation.

OAR	SMN In-Field Total(-CTV)											
Treatment Modality	3D-CRT		IMRT		SFUD		*p*-Value					
Dose Response Relation	Linear-Exponential (L-E)	Plateau (P)	Linear-Exponential	Plateau	Linear-Exponential	Plateau	3DCRT vs. IMRT		3DCRT vs. SFUD		IMRT vs. SFUD	
							L-E	P	L-E	P	L-E	P
Patient												
1	58.63	173.50	74.67	193.11	21.26	75.63	**	**	*	*	*	*
2	46.44	102.20	55.33	134.47	20.22	54.27	**	*	*	*	*	*
3	65.87	167.86	73.09	177.63	17.22	49.93	*	**	*	*	*	*
4	49.13	106.03	67.16	142.13	14.45	44.27	*	**	*	*	*	*
5	58.69	134.61	70.13	164.13	19.73	56.00	**	*	*	*	*	*
6	63.52	176.13	88.78	206.67	21.08	58.81	*	**	*	*	*	*
7	67.52	152.71	74.53	162.64	9.22	30.53	**	**	*	*	*	*
8	49.01	97.70	54.25	112.43	8.60	27.01	*	*	*	*	*	*
9	45.83	99.93	54.63	123.88	11.11	31.94	**	*	*	*	*	*
10	65.80	135.45	68.69	146.32	11.31	25.44	*	**	*	*	*	*
11	59.47	137.99	64.44	140.70	12.60	36.40	*	**	*	*	*	*
12	75.22	178.42	84.21	213.09	31.26	87.91	*	**	*	*	*	*

* *p* < 0.05, ** *p* > 0.05.

## Data Availability

The data presented in this study are available on request from the corresponding author. Data are taken from patient records and are therefore not publicly available.
